# Rapid Diagnosis of 83 Patients with Niemann Pick Type C Disease and Related Cholesterol Transport Disorders by Cholestantriol Screening^☆^

**DOI:** 10.1016/j.ebiom.2015.12.018

**Published:** 2015-12-22

**Authors:** Janine Reunert, Manfred Fobker, Frank Kannenberg, Ingrid Du Chesne, Maria Plate, Judith Wellhausen, Stephan Rust, Thorsten Marquardt

**Affiliations:** a Department of Pediatrics, University Hospital of Muenster, Albert-Schweitzer-Campus 1 Gebaeude A13, 48149 Muenster, Germany; b Center of Laboratory Medicine, University Hospital of Muenster, Albert-Schweitzer-Campus 1 Gebaeude A1, 48149 Muenster, Germany

**Keywords:** NPC, Niemann Pick type C, c-triol/cholestantriol, cholestane-3β,5α,6β-triol, 7-KC, 7-ketocholesterol, CESD, cholesterol ester storage disease, EVS, exome variant server, HGMD, Human Gene Mutation Database, ROC, receiver-operating-characteristic, Niemann Pick type C, Oxysterols, Cholestantriol, Diagnostic test

## Abstract

Niemann Pick type C (NP-C) is a rare neurodegenerative disorder caused by an impairment of intracellular lipid transport. Due to the heterogeneous clinical phenotype and the lack of a reliable blood test, diagnosis and therapy are often delayed for years. In the cell, accumulating cholesterol leads to increased formation of oxysterols that can be used as a powerful screening parameter for NP-C. In a large scale study, we evaluated the oxysterol cholestane-3β,5α,6β-triol (c-triol) as potential biomarker for a rapid diagnosis of NP-C. Using GC/MS, c-triol has been analyzed in 1902 plasma samples of patients with the suspicion for NP-C. Diagnosis in patients with elevated oxysterols was confirmed by genetic analysis. 71 new NP-C patients (69 NP-C1 and two NP-C2) and 12 Niemann Pick type A/B patients were identified. 24 new mutations in *NPC1*, one new mutation in *NPC2* and three new mutations in the *SMPD1* gene were found. Cholestane-3β,5α,6β-triol was elevated in Niemann Pick type C1, type C2, type A/B and in CESD disease. No other study has ever identified so many NP-C patients, proving that c-triol is a rapid and reliable biomarker to detect patients with NP-C disease and related cholesterol transport disorders. It should replace the filipin test as the first-line diagnostic assay.

## Highlights

• Improved diagnostics of Niemann Pick type C disease using a cholesterol oxidation product (c-triol) as biomarker
• Identification of 71 Niemann Pick type C patients by a simple blood test
• Cholestantriol should replace the filipin test as first-line diagnostic assay.

Due to the heterogeneous clinical phenotype and the lack of appropriate screening biomarkers, Niemann Pick type C was assumed to be an underdiagnosed disease. The accumulation of cholesterol and oxidative stress in NP-C cells lead to an increased oxidation rate of cholesterol. As already demonstrated by Porter et al. (2010) and Jiang et al. (2011), several oxysterols are elevated in confirmed NP-C patients. A large scale study was initiated to prove the clinical use of cholestane-3β,5α,6β-triol as a screening biomarker for Niemann Pick type C disease. We were able to identify numerous NP-C-patients in 1902 samples sent in from different countries, demonstrating that with appropriate screening centers much more patients would benefit from an earlier diagnosis and therapy.

## 1 Introduction

Niemann Pick type C (NP-C) is a neurovisceral disease that is caused by an impaired intracellular transport of cholesterol and glycolipids based on mutations in the *NPC1* or *NPC2* gene (Carstea et al., 1997, Naureckiene et al., 2000). NP-C is underdiagnosed and not readily identifiable due to a variable age of onset and a variety of age-dependent symptoms. Whereas younger patients present primarily with visceral symptoms such as hepatosplenomegaly followed by progressive intellectual and neurological deterioration, adults often develop psychiatric problems, including depression and psychosis (reviewed by Patterson et al., 2012, Mengel et al., 2013). Due to the heterogeneous clinical phenotype, diagnosis is often delayed for many years or missed altogether. Since a disease modifying therapy is available (Patterson et al., 2007) and more are being developed, there is an urgent need for a reliable and robust biomarker.

Interruption of cholesterol transport leads to an increased non-enzymatic oxidation of a very small fraction of the accumulated cholesterol in NP-C cells. The oxidation products of cholesterol, called oxysterols, can be measured by GC–MS or LC–MS/MS in human plasma. It has been shown that 7-ketocholesterol (7-KC) and cholestane-3β,5α,6β-triol (c-triol), are elevated in the plasma of NP-C1 and NP-C2 patients (Porter et al., 2010, Boenzi et al., 2014, Reunert et al., 2015, Jiang et al., 2011).

In a large scale investigator-initiated study, we evaluated c-triol as a potential biomarker for the diagnosis of Niemann Pick type C disease. Using GC–MS, 1902 plasma samples of patients with the suspicion of NP-C disease, carriers of a heterozygous mutation in the *NPC1* gene and confirmed NP-C patients were analyzed.

Our data demonstrate that analysis of plasma cholestane-3β,5α,6β-triol fulfills the need for a rapid and reliable biomarker for NP-C disease and related cholesterol transport disorders, making diagnosis and early therapy of these severe neurodegenerative disorders much easier.

## 2 Material and Methods

### 2.1 Patient Consents

Informed consent according to local laws was obtained from either the patient or their legal guardians by the physician in charge.

### 2.2 Sampling/Collection of Plasma Samples

2 ml EDTA-blood samples from patients suspected of having NP-C disease were collected. Samples of five confirmed NP-C1 patients, treated in our hospital, served as internal quality control. In order to include heterozygote carriers of NP-C mutations in the study, samples of parents and siblings of known NP-C patients were collected as well. If arrival in the laboratory within 48 h after drawing the blood was guaranteed, the sample was sent at room temperature. Otherwise, the plasma was separated from the cell pellet and both samples were sent on dry ice. Process of sample handling after receipt of the c-triol result can be seen in a flow chart in the supplementary material (Fig. S1).

### 2.3 Chitotriosidase Activity

The chitotriosidase activity was measured as previously described (Reunert et al., 2015, Hollak et al., 1994).

### 2.4 Mutation Analysis

In all samples with elevated plasma cholestane-3β,5α,6β-triol concentration, as well as in cases with strong clinical suspicion of NP-C but normal oxysterols, confirmatory molecular genetic analysis of the *NPC1* and *NPC2* genes was performed. The coding region of NPC1 (NM_000271) and NPC2 (NM_006432) and flanking intronic sequences were amplified by PCR and analyzed by Sanger sequencing. Putative mutations were confirmed by sequencing independent PCR products. In four cases, additional sequencing of the NPC1 or NPC2 transcript was necessary to confirm splicing defects. In positive samples that did not contain more than one mutated allele for *NPC1* or *NPC2*, a genetic analysis of the *SMPD1*, *GBA* and/or *LIPA* genes was performed. Primer sequences for *NPC1*, *NPC2*, and *SMPD1*, can be found in the supplement (Supplementary Table S4), and primer sequences for *GBA* and *LIPA* are available upon request. If available, parental samples were analyzed, confirming the segregation of the mutations.

### 2.5 GC–MS-analysis

100 μl plasma was used to quantify the cholestane-3β,5α,6β-triol concentration. As internal standard, 10 ng of d7-cholestane-3β,5α,6β-triol (Santa Cruz) was added. Measurement of cholestane-3β,5α,6β-triol concentrations was done as described (Reunert et al., 2015). The cut-off value was 50 ng/ml. In rare cases where only serum was available, serum instead of plasma was analyzed.

### 2.6 Role of the Funding Source

The funding organization did not play a role in design of the study, interpretation of data, preparation and submission of the manuscript. All authors had full access to all data in the study and the corresponding author had final responsibility for the decision to submit for publication.

## 3 Results

**Fig. 1:**
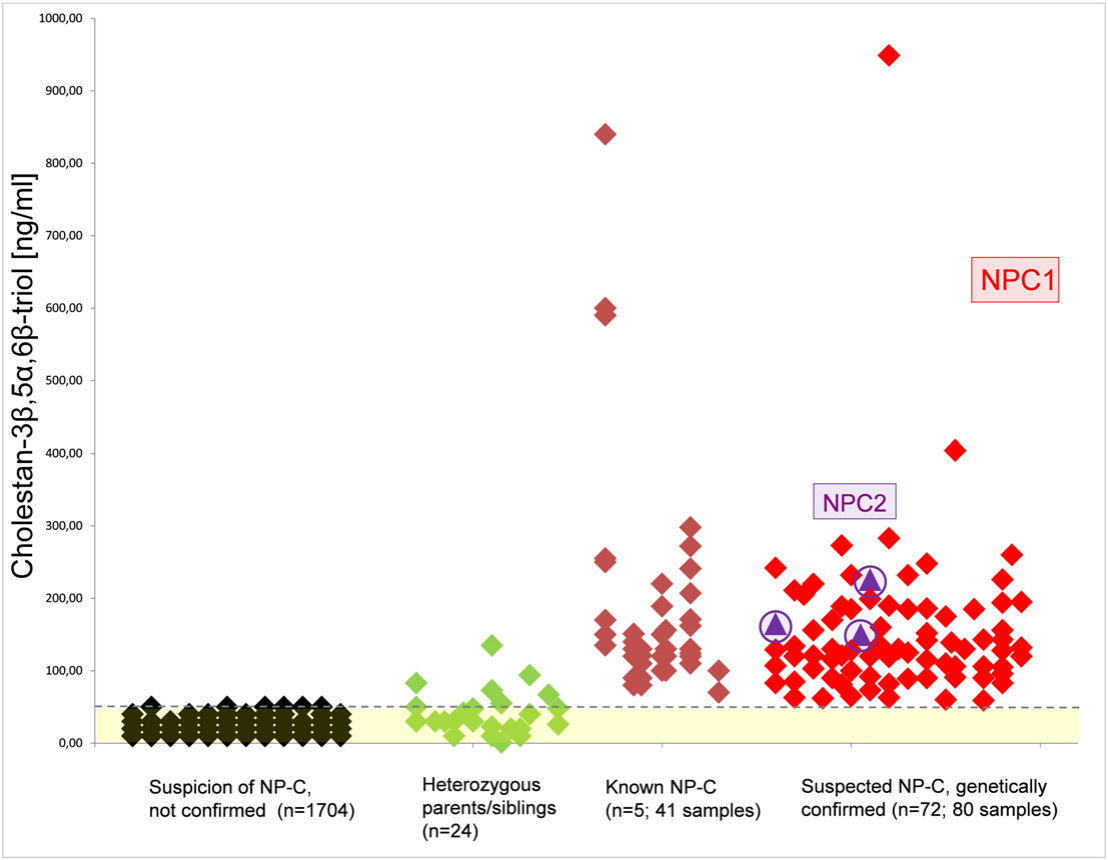
Cholestane-3β,5α,6β-triol-concentration in plasma of patients with suspicion of NP-C disease; yellow box marks the reference range, the dotted gray line indicates the cut-off value of 50 ng/ml; in the previously known NP-C patients, all rhombuses in a vertical column belong to the same patient (only for the known NP-C patients).

Within three years (2012–2014), cholestane-3β,5α,6β-triol was measured in 1902 plasma samples of patients with suspected NP-C disease (Fig. 1). 1704 samples had a normal c-triol concentration.

### 3.1 Heterozygotes

Six out of 24 confirmed carriers for a heterozygous mutation in NPC1 showed an increased c-triol concentration (s. Fig. 1). Three of these samples underwent sequencing of the *NPC1* and *NPC2* genes, but only the previously confirmed heterozygous mutation was found.

### 3.2 Previously Identified Patients

41 blood samples were drawn from five previously identified, genetically confirmed NP-C patients. Consecutive samples from patients were drawn upon routine follow-up visits in the hospital. All patients were on miglustat treatment. The c-triol concentration in these patients was in the range of 60 to 300 ng/ml, except for the first patient (s. Fig. 1). This patient presented for the first time at our hospital at the age of ten weeks, with cholestatic jaundice and a profound hepatosplenomegaly. The initial c-triol concentrations were massively increased up to 840 ng/ml. Although organomegaly did not improve with time, c-triol concentrations decreased reaching the same range as the other confirmed NP-C1 patients.

### 3.3 Newly Identified NP-C Patients

80 samples of 72 different patients showed an increased c-triol concentration (s. Fig. 1). Subsequent genetic analysis in these 72 patients revealed either a homozygous mutation or two compound heterozygous mutations in NPC1 (n = 69) or NPC2 (n = 3) (s. Supplementary Table S1). Three NP-C2 patients presented similar c-triol concentrations as 69 NP-C1 patients and could not be distinguished from the NP-C1 patients by c-triol analysis. One of these NP-C2 samples was a frozen serum sample, preserved for several years. It belonged to a deceased NP-C2 patient that had already been described by Griese et al. (2010). NPC-2 details have been published (Reunert et al., 2015).

C-triol concentrations, chitotriosidase activities, filipin staining results (if available) and results of genetic analysis of all patients are summarized in the Supplementary material (Table S1).

### 3.4 Specificity

33 patients have been false positive for NP-C. An elevated amount of plasma c-triol was detected in all of these patients (range 53–782 ng/ml), but in 30 patients no mutation in either NPC1 or NPC2 has been found; three patients were heterozygous for one mutation in NPC1. In only two patients, a second sample was available which also revealed a c-triol concentration > 50 ng/ml.

In 12 patients, additional genetic analyses revealed compound heterozygosity or a homozygous mutation in the *SMPD1* gene, identifying them as Niemann Pick type A/B patients (including two of the patients with one heterozygous mutation in NPC1). The diagnosis of the remaining patients is still unknown. In two patients with confirmed cholesterol ester storage disease (CESD) c-triol was also elevated (121 ng/ml–181 ng/ml). Analysis of the false positives for *LIPA* mutations did not reveal additional CESD patients. Further analysis of the *GBA* gene was done in two patients with a massively elevated chitotriosidase, as typically seen in Gaucher's disease, but did also not identify any mutations (see Supplement Table S3). In addition to the 33 false positive samples, there were 11 patients with an elevated c-triol concentration, where the second sample revealed normal values.

### 3.5 Sensitivity

In seven patients with normal c-triol concentrations, mutations in *NPC1* (n = 6) or *NPC2* (n = 1) were detected, confirming NPC disease. The chitotriosidase activity was elevated in three of these patients. Despite normal c-triol concentrations, a genetic analysis of *NPC1* or *NPC2* was performed, as these patients presented typical clinical symptoms, including ataxia, dysarthria, vertical supranuclear gaze palsy, dysphagia and psychosis (see Supplementary Table S2). In three of the seven patients, a second sample was available, which also revealed a c-triol concentration below 50 ng/ml. Two of the false negative NP-C patients showed a classical and three patients showed a variant staining pattern in filipin testing (see Supplementary Table S2).

### 3.6 Sensitivity and Specificity

ROC analysis of 1811 samples was applied using MedCalc software. With a cut-off of 50 ng/ml, a sensitivity of 91.8% and a specificity of 97.5% were determined.

### 3.7 Correlation of C-triol with Age of Patient/Onset

**Fig. 2:**
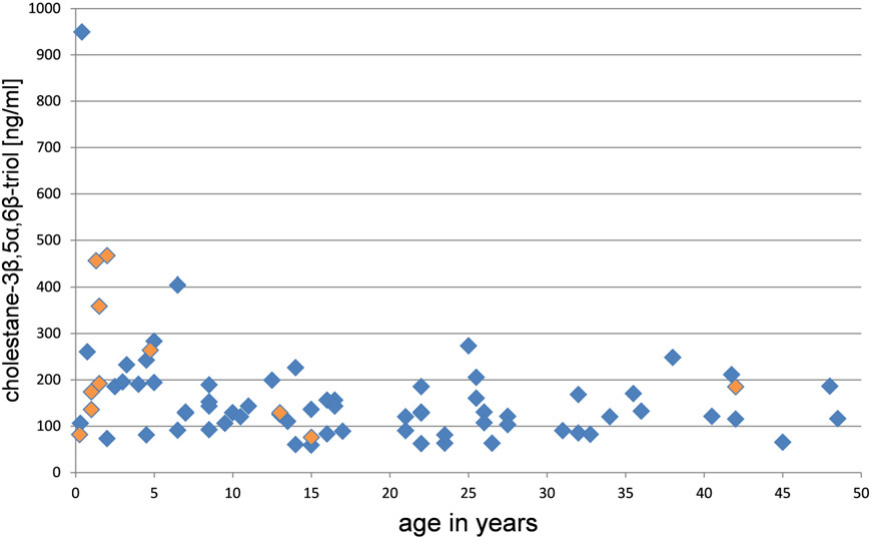
Analysis of c-triol with respect to the age of patient/onset in the newly identified patients. Blue rhombuses represent NP-C1 patients; orange rhombuses represent NP-A/B patients.

When the c-triol concentration was analyzed with respect to the age of the patients, only infantile patients with profound organomegaly had higher values. In other age-groups, the concentration of c-triol was age-independent (s. Fig. 2). Assuming that a more severe burden of disease would lead to an earlier diagnosis, higher oxysterol concentrations with younger age could have been expected. However, this was not found.

### 3.8 Monitoring Disease Therapy

**Fig. 3:**
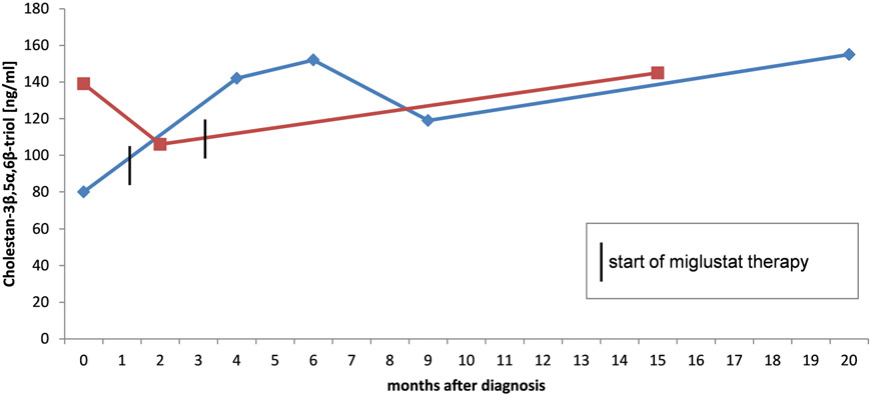
Course of c-triol concentrations in two NP-C1 patients before and during miglustat therapy; red curve = nine year old patient presenting visceral and psychiatric symptoms; blue curve = eight year old patient presenting visceral and neurological symptoms.

In two patients, aged 8 and 9 years, c-triol was analyzed before and during miglustat therapy. The eight year old patient mainly showed visceral (splenomegaly) and neurological symptoms (e.g. frequent falls). The nine year old boy presented with visceral (splenomegaly) and psychiatric symptoms (ADHD). During therapy, the progression of the disease in both patients was largely stable. C-triol concentrations did not decrease with miglustat treatment (s. Fig. 3).

### 3.9 Genetic Analyses

23 new mutations in *NPC1* were identified (s. Table 1), 13 missense mutations (C97S, F101C, N169I, L176R, G343E, E391K, P733R, M866T, V920G, V950G, S1004P, L1045P, A1187G), one nonsense mutation (R348X), four splice-site mutations (IVS13 + 2T > C, IVS20 + 5G > A, IVS24 + 1G > A, IVS24 + 3A > C) and five insertions or deletions (c.1182insT, c.1448_c.1449delGT, c.1654_1655insG, c.2683insG, c.3458insTC) leading to a frameshift and a premature stop codon. In the *NPC2* gene, two homozygous splice site mutations were identified, IVS3 + 6T > G which leads to the skipping of the complete exon 3 (confirmed by sequencing the transcript) and IVS4 + 1G > A, a known rare variant (rs140130028). In *SMPD1*, three new mutations were identified (C91H, L121P, Y374X) (s. Table 1). All newly described mutations have been predicted to be disease causing by the online prediction program “mutation taster” (Schwarz et al., 2014) and were not found in the EVS (exome variant server) and HGMD (Human Gene Mutation Database). An additional analysis by the prediction program provean, also predicted a “deleterious” outcome for the missense mutations (Choi et al., 2012).

### 3.10 Stability with Storage and Shipping

**Fig. 4:**
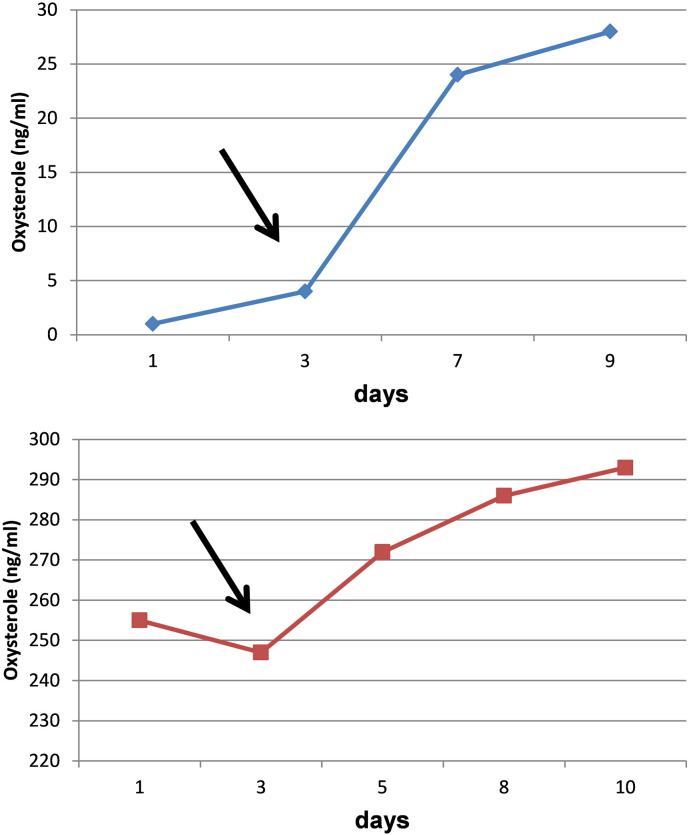
Stability of cholestane-3β,5α,6β-triol at room temperature. Blue curve: healthy control; red curve: confirmed NP-C1 patient. After 72 h of incubation at room temperature, the concentration of c-triol increases in both samples.

In order to examine the stability of c-triol at room temperature, plasma samples from two confirmed NP-C1 patients and from a healthy control have been analyzed at different time points after storage at room temperature. The concentration of plasma c-triol increased over time, starting at 72 h after drawing the blood. The healthy control presented a c-triol concentration near the cut-off after nine days (s. Fig. 4).

## 4 Discussion

The lack of appropriate biomarkers for the diagnosis of Niemann Pick type C leads to an underestimation of the incidence of this disease which is thought to be 1:120,000–150,000 in Western Europeans (Vanier and Millat, 2003). Indeed, Wassif et al. (2015) estimated the combined incidence of NP-C1 and NP-C2 as 1:89,000. In the current study, 71 new NP-C patients were diagnosed by oxysterol analysis followed by genetic analysis in a time frame of three years (2012–2014). Additionally, seven NP-C patients were identified by genetic analysis combined with filipin staining or high clinical suspicion. 25% of the heterozygote carriers (6/24) had c-triol levels above the cut-off value. This is exactly the same frequency of positive carriers as reported by Jiang et al. (2011) which shows the consistency of the assay across different platforms and centers.

Due to the clinical heterogeneity, NP-C patients usually endure an average diagnostic delay of 5–6 years (Sévin et al., 2007, Wraith and Imrie, 2009, Stampfer et al., 2013). A reliable biomarker that is effective at all ages and at all stages of the disease would be a benefit for physicians and patients. By measuring c-triol in plasma, we were able to identify NP-C patients from the age of 0.3–48.5 years, with variable neurological involvement, showing that oxysterols, or at least c-triol, are suitable as biomarker where age of onset and the degree of neurological involvement are irrelevant. As a modifying therapy is available for Niemann Pick type C, the time to diagnosis is important allowing earlier treatment. All NP-C1 patients identified prior to the study were treated with miglustat. As expected, no effect of the sphingolipid synthesis inhibitor miglustat on the c-triol concentration was obvious which indicates that the measurement of c-triol cannot be used to monitor miglustat treatment effects in NP-C patients.

Porter et al. (2010) stated that plasma oxysterol concentrations correlated with disease severity and age of onset. In our study, this was not confirmed in patients beyond infancy. In children with an infantile onset, the c-triol concentration was higher than in juvenile and adult onset of the disease. Two newborn children with massive organomegaly presented the highest c-triol concentrations measured in this study. In one of them, the plasma c-triol decreased in the first year of life. The other child died after a few months.

The practicability of storage and shipping of the samples is an important factor considering a reliable result. Sending samples on dry ice would be the first choice to maintain sample stability and avoid autoxidation of the cholesterol, but is impractical and often impossible for small hospitals. We show that after 72–96 h at room temperature, the concentration of c-triol increased in the NP-C1 samples and in the healthy control. Considering that the sample of the NPC1 patient has already been pathological before incubation at RT, the elevation from 255 to 293 ng/ml in ten days is negligible. However, the initial c-triol concentration of the healthy control was 1 ng/ml and increased to 28 ng/ml in nine days. Other healthy controls showed c-triol concentrations of 10–30 ng/ml and would have reached the cut-off shortly after 72 h at room temperature. We recommend sending samples for c-triol-analysis at room temperature when it can be guaranteed that they arrive in the laboratory within 48 h. If the shipping time lasts more than 48 h, a negative c-triol concentration could shortly reach the cut-off. This would present a significant risk of false positive samples, which includes further unnecessary analyses, e.g. genetic analyses or a skin biopsy followed by filipin staining. Additionally, hemolysis should be avoided as samples with hemolysis prior to centrifugation also give false positive results (unpublished data; manuscript submitted by Frank Kannenberg, Jerzy-Roch Nofer, Erhard Schulte, Janine Reunert, Thorsten Marquardt, Manfred Fobker).

33 positive samples with only one or without mutations in the *NPC1* or *NPC2* gene were detected in this study. These false positive samples did not exclusively arise by wrong shipping or storage conditions or due to hemolysis. In 12 of these samples, mutations in the *SMPD1* gene were found. These data show that c-triol is also increased in Niemann Pick type A/B patients. Lin et al. (2014), had shown that another oxysterol, 7-ketocholesterol (7-KC) is also increased in Niemann Pick type A/B patients. There are still several positive samples left where the diagnosis is not known yet. We suggest that there might be other diseases where a higher oxidation of the cholesterol occurs. Investigation in two CESD patients (cholesterol ester storage disease) also revealed higher concentrations of plasma c-triol. Most of the remaining positive samples were screened for mutations in the *LIPA* gene, but no mutation was found. In these cases we consider transcript analysis and/or whole exome sequencing as next diagnostic steps. In the samples of 11 patients with elevated c-triol concentrations, a consecutive sample showed normal results. In most of these cases the shipping duration of the first sample exceeded the recommended 48 h. In seven c-triol-negative samples, a genetic analysis revealed disease causing mutations in the *NPC1* or *NPC2* gene. The reason why there are clinically and genetically confirmed NP-C patients with a normal c-triol concentration is not known yet and has to be further investigated. There might be more potential false negative NP-C patients hidden in the cohort of samples that were below the cut-off value, as a genetic analysis of *NPC1* and *NPC2* was not usually performed in all samples. Jiang et al. (2011) also reported 2.7% of NP-C patients that were below the cut-off for their assay.

Several new mutations in the *NPC1*, *NPC2* and *SMPD1* genes were found in this study. All these mutations are predicted to be disease causing (online program “mutation taster” Schwarz et al., 2014) except for the splice mutation IVS4 + 1G > A in *NPC2*, which is a known variant (rs140130028). Only heterozygote patients with this mutation have been described in the literature (Bauer et al., 2013), already presenting symptoms of NPC disease. However, while the frequency of this variant in the population was not mentioned in that paper, indeed it was similar to the frequency of the mutation in the NPC-patient group, and therefore heterozygosity for this mutation was most likely not causative for the phenotype. Patient 73 carries the IVS4 + 1G > A mutation in a homozygous state and shows symptoms typical for NPC (see Table S2). Further investigations on this patient and the splice mutation have been initiated. The most common NPC1 mutations among western Europeans, I1061T and P1007A, were found in 13/150 alleles (I1061T) and 23/150 alleles (P1007A) respectively (I1061T: 3/13 South America, 10/13 Europe; P1007A: 3/24 South America, 20/24 Europe).

In patients 84.1 and 84.2, one heterozygote mutation was found in the *NPC1* gene. After unsuccessful extensive sequencing, the second disease causing mutation could not be found and NP-C1 has been excluded as suspected diagnosis. Due to the elevated c-triol concentration that was also found in NP-A/B patients, the *SMPD1* gene was sequenced and two known heterozygous mutations have been found. The presence of one heterozygous mutation was puzzling and led to a diagnostic delay.

However, in other cases deep intronic mutations that escape when sequencing only exons and exon/intron-boundaries may lead to alternative splicing, instable mRNA, etc. effects that are only discovered by complex further analyses e.g. of cDNA.

Other biomarkers are tested in parallel to the oxysterols, including bile acids in urine and plasma parameters such as lysosphingolipids or CCL18/PARC. In retrospective studies it has been shown that lysosphingolipids and CCL18/PARC are also elevated in NP-C patients (Welford et al., 2014, Chang et al., 2010). There are no prospective studies yet that show the clinical use of these promising parameters as potential biomarkers for NP-C.

No other study has described the identification of so many new patients in such a short time frame. Oxysterol analysis is a rapid and reliable assay for screening of patients suspected to have NP-C disease and should replace the filipin test as the first-line diagnostic assay. Still, filipin staining should be performed in uncertain cases.

## 5 Conclusions

Our data demonstrate the clinical use of c-triol as potential biomarker for a rapid diagnosis of Niemann Pick type C disease. Measuring c-triol in human plasma is cost-effective, is less invasive than other traditional tests and might lead to a diagnosis at an early stage of the disease. We show that c-triol is elevated in NP-C1, NP-C2, NP-A/B, and CESD patients.

## Declaration of Interests

JR, FK and TM have received travel reimbursements and speaker honorarium from Actelion Pharmaceuticals Ltd., Allschwil, Switzerland.

SR has received travel reimbursements from Actelion Pharmaceuticals Ltd., Allschwil, Switzerland.

MF, JW, ID and MP declare that they have no conflict of interest.

## Author Contributions

JR: acquisition and interpretation of data, drafting and revising the manuscript.

FK, MF: acquisition and interpretation of c-triol data and revising the manuscript.

IDC, MP, JW: acquisition of data and revising the manuscript.

TM: supervising and design of the study, interpretation of data, and revising the manuscript.

## Figures and Tables

**Table 1:** Summary of newly identified mutations in *NPC1*, *NPC2* and *SMPD1*.

Exon/intron	Nucleotide mutation (+ 1 as A of ATG start codon)	Consequence of mutation	Mutation type
*NPC1*			
4	c.289T > A	C97S	Missense
4	c.302T > G	F101C	Missense
5	c.506A > T	N169I	Missense
5	c.527T > G	L176R	Missense
8	c.1028G > A	G343E	Missense
8	c.1042C > T	R348X	Nonsense
8	c.1171G > A	E391K	Missense
8	c.1182insT	fs396X	Frameshift
9	c.1448_c.1449delGT	fs524X	Frameshift
10	c.1654_1655insG	fs557X	Nonsense
IVS13	IVS13 + 2T > C		Splicing
14	c.2198C > G	P733R	Missense
17	c.2597T > C	M866T	Missense
18	c.2683insG	fs917X	Frameshift
18	c.2759T > G	V920G	Missense
19	c.2849T > G	V950G	Missense
20	c.3010T > C	S1004P	Missense
IVS20	IVS20 + 5G > A		Splicing
21	c.3134T > C	L1045P	Missense
22	c.3458insTC	fs1159X	Frameshift
23	c.3560C > G	A1187G	Missense
IVS24	IVS24 + 1G > A^a^		Splicing
IVS24	IVS24 + 3A > C		Splicing

*NPC2*			
IVS3	IVS3 + 6T > G	ex3del/fs75X	Splicing

*SMPD1*			
1	c.271_272delinsCA	C91H	Missense
2	c.362T > C	L121P	Missense
3	c.1122C > A	Y374X	Nonsense

a IVS24 + 1G > C (Millat et al., 2001).
