# Supplementary material for: Rapid Diagnosis of 83 Patients with Niemann Pick Type C Disease and Related Cholesterol Transport Disorders by Cholestantriol Screening
Source: eBioMedicine. 2015 Dec 22;4:170–5. doi: 10.1016/j.ebiom.2015.12.018 (PMC4776073; doi:10.1016/j.ebiom.2015.12.018)
Supplement: Supplementary file 1 — Supplementary material. [file mmc1.pdf]

Supplementary information

Rapid diagnosis of 83 patients with Niemann Pick type C disease and related cholesterol transport disorders by cholestantriol screening

Janine Reunert PhD[1], Manfred Fobker PhD[2], Frank Kannenberg PhD[2], Ingrid Du Chesne[1], Maria Plate[1], Judith Wellhausen, Stephan Rust PhD[1], Thorsten Marquardt MD[1§]

[1]Department of Pediatrics, University Hospital of Muenster, Muenster, Germany

Albert-Schweitzer-Campus 1 Gebaeude A13, 48149 Muenster, Germany

[2]Center of Laboratory Medicine, University Hospital of Muenster, Muenster, Germany

Albert-Schweitzer-Campus 1 Gebaeude A1, 48149 Muenster, Germany

[§]Corresponding author: Address: Klinik für Kinder- und Jugendmedizin, Albert-Schweitzer-Campus 1 A13, 48149 Muenster, Germany, Phone: +49 251 835 6494, Fax: +49 251 835 6085, Email address: marquat@uni-muenster.de

Content

1. **Supplementary tables**
   - **Supplementary table S1** Summary of c-triol concentrations, chitotriosidase activities, filipin staining and results of genetic analysis of all new patients that were identified during the study
   - **Supplementary table S2** Summary of clinical symptoms and biochemical results in false negative NP-C1 patients
   - **Supplementary figure S1** Process of sample handling after receiving the sample of a patient with the suspicion of NP-C disease.
   - **Supplementary table S3** Enhanced molecular genetic analyses in false positive patients
   - **Supplementary table S4** Primer sequences of NPC1, NPC2 (gDNA and transcript) and SMPD1 (gDNA)

Supplementary table 1 Summary of c-triol concentrations, chitotriosidase activities, filipin staining and results of genetic analyses of all new patients that were identified during the study

**patient**	**subject age at sampling (yrs)**	**cholestantriol ng/ml (normal<50)**	**chitotriosidase nmol/h/ml (normal<100)**	**filipin staining**	**Consequence of mutation**	**Nucleotide mutation (+1 as A of ATG start codon)**	**Reference**
**NP-C1**							
1	26	130	817		S940L<br>S940L	c.2819C>T<br>c.2819C>T	Greer et al., 1999[1]<br>Greer et al., 1999[1]
2	7	128	585		S357L<br>S940L	c.1070C>T<br>c.2819C>T	Saito et al., 2002[2]<br>Greer et al., 1999[1]
3.1	7	130	<10		A927V<br>S1004P	c.2780C>T<br>c.3010T>C	Meiner et al., 2001[3]<br>**this study**
3.2	4	190	203		A927V<br>S1004P	c.2780C>T<br>c.3010T>C	Meiner et al., 2001[3]<br>**this study**
4	34	120	615		I1061T<br>R1077Q	c.3182T>C<br>c.3230G>A	Millat et al.,1999[4] / Yamamoto et al., 1999[5]<br>Fancello et al., 2009[6]
5	21	120	106		C97S<br>S954L	c.289T>A<br>c.2861C>T	Watari et al., 1999[7] / **this study**<br>Greer et al., 1999[1]
6.1	22	130	217		S954L<br>fs1005x	c.2861C>T<br>c.2972_2973delAG	Greer et al., 1999[1]<br>Greer et al., 1999[1]
7	8.5	152	1480		V950G<br>I1061T	c.2849T>G<br>c.3182T>C	**this study**<br>Millat et al.,1999[4] / Yamamoto et al., 1999[5]
8	0.75	260	518		R348X<br>S954L	c.1042C>T<br>c.2861C>T	**this study**<br>Greer et al., 1999[1]
9	21	90	470		fs396X<br>S954L	c.1182insT<br>c.2861C>T	**this study**<br>Greer et al., 1999[1]
10.1	22	185	99		N1156S<br>N1156S	c.3467A>G<br>c.3467A>G	Carstea et al., 1997[8]<br>Carstea et al., 1997[8]

10.2	13.5	110	888.2		N1156S<br>N1156S	c.3467A>G<br>c.3467A>G	Carstea et al., 1997[8]<br>Carstea et al., 1997[8]
11	22	62	214		P1007A<br>A1035V	c.3019C>G<br>c.3104C>T	Greer et al., 1999[1]<br>Ribeiro et al., 2001[9]
12	25.5	205	75.2		P401T<br>P1007A	c.1201C>A<br>c.3019C>G	Sun et al., 2001[10]<br>Greer et al., 1999[1]
13.1	16.5	156	309		IVS13+2T>C<br>P1007A	IVS13+2T>C<br>c.3019C>G	**this study**<br>Greer et al., 1999[1]
13.2	8.5	92	456.5		IVS13+2T>C<br>P1007A	IVS13+2T>C<br>c.3019C>G	**this study**<br>Greer et al., 1999[1]
14	6.5	404	<10		fs126X<br>M866T	c.351delGA<br>c.2597T>C	Yamamoto et al., 1999[5]<br>**this study**
15	25.5	160	174		L1045P<br>A1151T	c.3134T>C<br>c.3451G>A	**this study**<br>Garver et al., 2010[11]
16	15	136	44.5		N169I<br>N169I	c.506A>T<br>c.506A>T	**this study**<br>**this study**
17	25	273	174		A764A<br>G1140V	c.2292G>A<br>c.3419G>T	Park et al., 2003[12]<br>Park et al., 2003[12]
18	4.5	81	279		fs1005X<br>P1007A	c.2972_2973delAG<br>c.3019C>G	Greer et al., 1999[1]<br>Greer et al., 1999[1]
19	8.5	189	831		I1061T<br>I1061T	c.3182T>C<br>c.3182T>C	Millat et al.,1999[4] / Yamamoto et al., 1999[5]<br>Millat et al.,1999[4] / Yamamoto et al., 1999[5]
20	9.5	106	88.9		S954L<br>R1186H	c.2861C>T<br>c.3557G>A	Greer et al., 1999[1]<br>Carstea et al., 1997[8]
21	6.5	91	1535	negative	Y1019C<br>c.1554-1009G>A	c.3056<br>c.1554-1009G>A	Fancello et al., 2009[6]<br>Rodríguez-Pascau et al., 2009[13]
22	na	119	<10	positive	A764A<br>R958X	c.2292G>A<br>c.2872C>T	Park et al., 2003[12]<br>Sun et al., 2001[10]
23	14	60	103		S954L<br>IVS20+5G>A	c.2861C>T<br>IVS20+5G>A	Greer et al., 1999[1]<br>**this study**

24	36	132	392.5		V920G<br>G992R	c.2759T>G<br>c.2974G>C	**this study**<br>Greer et al., 1998[14]
25	2.5	212	310,1		fs557X<br>fs557X	c.1654_1655insG<br>c.1654_1655insG	**this study**<br>**this study**
26	2	73	128		P887L<br>I1061T	c.2660C>T<br>c.3182T>C	Garver et al., 2010[11]<br>Millat et al.,1999[4] / Yamamoto et al., 1999[5]
27	2.5	185	1166		D944N<br>T1036M	c.2830G>A<br>c.3107C>T	Millat et al., 2001[15]<br>Carstea et al., 1997[8]
28	12.5	199	589	positve	R404W<br>M1001V	c.1210C>T<br>c.3001A>G	Park et al., 2003[12]<br>Stampfer et al., 2013[16]
29	23.5	63	195.4		P1007A<br>P1007A	c.3019C>G<br>c.3019C>G	Greer et al., 1999[1]<br>Greer et al., 1999[1]
30.1	45	65	110.4		P1007A<br>P1007A	c.3019C>G<br>c.3019C>G	Greer et al., 1999[1]<br>Greer et al., 1999[1]
30.2	48	186	158.7		P1007A<br>P1007A	c.3019C>G<br>c.3019C>G	Greer et al., 1999[1]<br>Greer et al., 1999[1]
31	16	156	<10		IVS7+5G>A<br>P1007A	IVS7+5G>A<br>c.3019C>G	Stampfer et al., 2013[16]<br>Greer et al., 1999[1]
32	27.5	121	51.8		S954L<br>R1186H	c.2861C>T<br>c.3557G>A	Greer et al., 1999[1]<br>Carstea et al., 1997[8]
33	17	89	73.1		P1007A<br>I1061T	c.3019C>G<br>c.3182T>C	Greer et al., 1999[1]<br>Millat et al.,1999[4] / Yamamoto et al., 1999[5]
34	13	125	52		F101C<br>IVS24+1G>A	c.302T>G<br>IVS24+1G>A	**this study**<br>**this study***
35	42	115	815.5		P434L<br>I1061T	c.1301C>T<br>c.3182T>C	Fernandez-Valero et al., 2005[17]<br>Millat et al.,1999[4] / Yamamoto et al., 1999[5]
36	0.3	106	205.6		I1061T<br>fs1241X	c.3182T>C<br>c.3618delA	Millat et al.,1999[4] / Yamamoto et al., 1999[5]<br>Yamamoto et al., 2000[18]
37	16.5	143	67.6		A764A<br>Q775P	c.2292G>A<br>c.2324A>C	Park et al., 2003[12]<br>Millat et al., 2001[15]

38	15	59	52	positive	A764A<br>A1035V	c.2292G>A<br>c.3104C>T	Park et al., 2003[12]<br>Ribeiro et al., 2001[9]
39.1	10	129	349		P1007A<br>fs1240X	c.3019C>G<br>c.3611_3614delTTAC	Greer et al., 1999[1]<br>Park et al., 2003[12]
39.2	8.5	143	1472.9		P1007A<br>fs1240X	c.3019C>G<br>c.3611_3614delTTAC	Greer et al., 1999[1]<br>Park et al., 2003[12]
40	11	143	792	variant	A1035V<br>I1061T	c.3104C>T<br>c.3182T>C	Ribeiro et al., 2001[9]<br>Millat et al.,1999[4] / Yamamoto et al., 1999[5]
41	32	85	43		V664M<br>I1061T	c.1990G>A<br>c.3182T>C	Park et al., 2003[12]<br>Millat et al.,1999[4] / Yamamoto et al., 1999[5]
42	41.75	211	457.9		N968S<br>A1151T	c.2903A>G<br>c.3451G>A	Yang et al., 2005[19]<br>Garver et al., 2010[11]
43	26	107	201		S940L<br>S954L	c.2819C>T<br>c. 2861C>T	Greer et al., 1999[1]<br>Greer et al., 1999[1]
44	4.5	242	922		R404Q<br>IVS24+3A>C	c.1211G>A<br>IVS24+3A>C	Millat et al.,2001[15]<br>**this study**
45	na	96	113.9		C664M<br>C664M	c.1990G>A<br>c.1990G>A	Park et al., 2003[12]<br>Park et al., 2003[12]
46	5	194	520	positive	R1186H<br>R1186H	c.3557G>A<br>c.3557G>A	Carstea et al., 1997[8]<br>Carstea et al., 1997[8]
47	na	220	na		Y825C<br>I1061T	c.2474A>G<br>c.3182T>C	Millat et al., 2001[15]<br>Millat et al.,1999[4] / Yamamoto et al., 1999[5]
48	16	83	593	positive	V664M<br>A1035V	c.1990G>A<br>c.3104C>T	Park et al., 2003[12]<br>Ribeiro et al., 2001[9]
49	22	128	<10	variant	fs917X<br>P1007A	c.2683insG<br>c.3019C>G	**this study**<br>Greer et al., 1999[1]
50	14	226	885.6		I1061T<br>R1186H	c.3182T>C<br>c.3557G>A	Millat et al.,1999[4] / Yamamoto et al., 1999[5]<br>Carstea et al., 1997[8]
51	10.5	120	588		L176R<br>P1007A	c.527T>G<br>c.3019C>G	**this study**<br>Greer et al., 1999[1]

52	3	195	1213.6		fs1005X<br>fs1005X	c.2972_c.2973delAG<br>c.2972_c.2973delAG	Greer et al., 1999[1]<br>Greer et al., 1999[1]
53	40.5	121	145		S954L<br>P1007A	c.2861C>T<br>c.3019C>G	Greer et al., 1999[1]<br>Greer et al., 1999[1]
54	27.5	103	115.4		A764A<br>I1061T	c.2292G>A<br>c.3182T>C	Park et al., 2003[12]<br>Millat et al.,1999[4] / Yamamoto et al., 1999[5]
55	32	168	88.3		G343E<br>P733R	c.1028G>A<br>c.2198C>G	**this study**<br>**this study**
56	48.5	116	<10		S954L<br>P1007A	c.2861C>T<br>c.3019C>G	Greer et al., 1999[1]<br>Greer et al., 1999[1]
57	3.25	232	140.3		fs1005X<br>fs1005X	c.2972_c.2973delAG<br>c.2972_c.2973delAG	Greer et al., 1999[1]<br>Greer et al., 1999[1]
58	0.4	949	215.4		R116X<br>E391K	c.346C>T<br>c.1171G>A	Kaminski et al., 2002[20]<br>**this study**
59	32.75	82	62.3		P474L<br>P1007A	c.1421C>T<br>c.3019C>G	Tarugi et al., 2002[21]<br>Greer et al., 1999[1]
60	31	90	464.6		I1061T<br>N1156S	c.3182T>C<br>c.3467A>G	Millat et al.,1999[4] / Yamamoto et al., 1999[5]<br>Carstea et al., 1997[8]
61	5	283	639.5		fs524X<br>Q775P	c.1448_c.1449delGT<br>c.2324A>C	**this study**<br>Millat et al.,2001[15]
62	38	248	108.4		P1007A<br>P1007A	c.3019C>G<br>c.3019C>G	Greer et al., 1999[1]<br>Greer et al., 1999[1]
63	23.5	81	169.2		L684F<br>A1187G	c.2050C>T<br>c.3560C>G	Park et al., 2003[12]<br>**this study**
64	26.5	63	67.5		R348X<br>V1165M	c.1042C>T<br>c.3493G>A	**this study**<br>Sun et al., 2001[10]; Park et al., 2003[12]
**NP-C2**							
65	0.75	150	415		E118X<br>E118X	c.352G>T<br>c.352G>T	Millat et al., 2001b[22]<br>Millat et al., 2001b[22]

66	na	226	<10		frame shift, elongated protein with additional 80 aa	c.408_409delAA<br>c.408_409delAA	Griese et al., 2010[23]<br>Griese et al., 2010[23]
67	7	130	169.4		ex3del/fs75X<br>ex3del/fs75X	IVS3+6T>G<br>IVS3+6T>G	**this study**<br>**this study**
**false negative NP-C1**							
68	27.5	30	25.8		S954L<br>W1145R	c.2861C>T<br>c.3433T>C	Greer et al., 1999[1]<br>Stampfer et al., 2013[16]
6.2	23.5	30	244	positive	S954L<br>fs1005X	c.2861C>T<br>c.2972_2973delAG	Greer et al., 1999[1]<br>Greer et al., 1999[1]
69	72.5	40	37.9	positive	V378A<br>V664M	c.1133T>C<br>c.1990G>A	Millat et al., 2001[15]<br>Park et al., 2003[12]
70	60.5	20	30.5		D611G<br>V1158M	c.1832A>G<br>c.3472G>A	Zhang et al., 2014[24]<br>Zech et al., 2013[25]
71	17.5	20	239		P1007A<br>fs1159X	c.3019C>G<br>c.3458insTC	Greer et al., 1999[1]<br>**this study**
72	27	44	88.4	positive (variant)	A764A<br>Q775P	c.2292G>A<br>c.2324A>C	Park et al., 2003[12]<br>Millat et al., 2001[15]
**false negative NP-C2**							
73	18.5	10	108	variant	IVS4+1G>A<br>IVS4+1G>A	IVS4+1G>A<br>IVS4+1G>A	**this study/** Bauer et al., 2013[26]<br>**this study/**Bauer et al., 2013[26]
**NP-A/B**							
74	42	185	1380		G244R<br>IVS3+3del4	c.730G>A<br>IVS3+3del4	Takahashi et al., 1992[27]<br>Rodriguez-Pascau et al., 2009b[28]
75	1.3	456	2539		C91H<br>C91H	c.271_272delinsCA<br>c.271_272delinsCA	**this study**<br>**this study**
76	1	136	<10		H423Y<br>H423Y	c.1267C>T<br>c.1267C>T	Simonaro et al., 2002[29]<br>Simonaro et al., 2002[29]

77	4.75	264	195.5		L121P<br>L121P	c.362T>C<br>c.362T>C	**this study**<br>**this study**
78	1	174	<10		L121P<br>L121P	c.362T>C<br>c.362T>C	**this study**<br>**this study**
79	2	467	2017.9		Y448C<br>Y448C	c.1343A>G<br>c.1343A>G	Takahashi et al., 1995[30]<br>Takahashi et al., 1995[30]
80	na	329	228	negative	fsR610del<br>fsR610del	c.1829_1831delGCC<br>c.1829_1831delGCC	**this study**<br>**this study**
81	1.5	359	700.6		Y374X<br>Y374X	c.1122C>A<br>c.1122C>A	**this study**<br>**this study**
82	1.5	192	619		Y448C<br>F482L	c.1343A>G<br>c.1446C>A	Takahashi et al., 1995[30]<br>Simonaro et al., 2002[29]
83	0.25	82	148.2		Y374X<br>Y374X	c.1122C>A<br>c.1122C>A	**this study**<br>**this study**
84.1	15	76	149		R291H<br>Q294K	c.872G>A<br>c.880C>A	Simonaro et al., 2002[29]<br>Pavlu and Elleder 1997[31]
84.2	13	129	571		R291H<br>Q294K	c.872G>A<br>c.880C>A	Simonaro et al., 2002[29]<br>Pavlu et al., 1997[31]
siblings are represented as 3.1 and 3.2, 6.1 and 6.2 etc.							
<10: no activity, suspected duplication in the chitotriosidase gene							
*IVS24+1G>C described by Millat et al., 2001							

Supplementary table S2 Summary of clinical symptoms and biochemical results in false negative NP-C1 patients

	patient No.						
**symptoms**	**68**	**6.2**	**69**	**70**	**71**	**72**	**73**
neonatal cholestatic hyperbilirubinemia	-	-	na	na	na	na	-
splenomegaly	+	-	+	na	-	na	-
vertical supranuclear gaze palsy	+	+	-	na	na	na	+
gelastic cataplexy	-	-	-	na	+	na	-
ataxia, clumsiness or frequent falls	+	+	-	na	+	na	+
dysarthria	+	+	-	na	na	na	+
loss of developmental milestones	+	-	-	na	+	na	+
seizures (partial or generalised)	-	-	-	na	+	na	-
other	psychosis	two episodes of psychosis, severe dysphagia	hypercholesterolemia, type 2 diabetes, lipid-laden macrophages in the spleen	na	cachexia, severe dysphagia, loss of deliberate communication	na	dysphagia
filipin staining	na	positive	positive	variant	na	variant	variant
c-triol 1. sample (ng/ml)	30	30	42	20	20	44	8
c-triol 2. sample (ng/ml)	20	na	40	na	na	na	11

Supplementary figure 1 Process of sample handling after receiving the sample of a patient with the suspicion of NP-C disease.

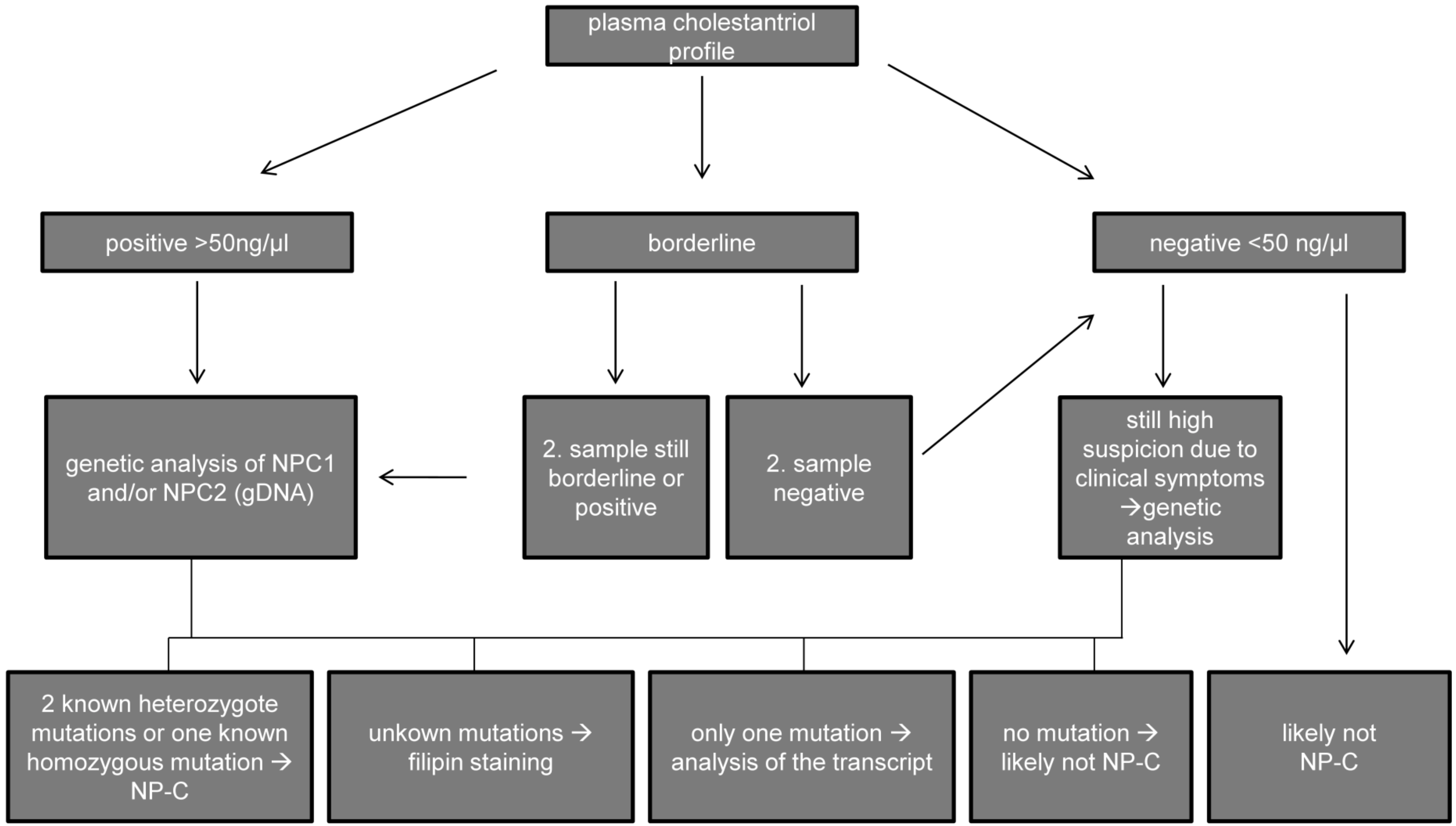

Supplementary table 3 Enhanced molecular genetic analyses in false positive patients

patient	subject age at sampling (yrs)	cholestantriol ng/ml (normal<50)	SMPD1	LIPA	GBA
**NPC1/2 neg**					
85	1.5	782	neg	neg	
86	23.5	64			
87	62.75	62	neg		
88	5.75	597	neg	1 het Stop	neg
89	66	55		neg	
90	51.5	86	neg	neg	
91	69.5	53			
92	17.75	79			
93	47	77			
94	3	255	neg	neg	neg
95	44.25	215			
96	37.75	59			
97	0.16	67			
98	71	84	neg		
99	32	75			
100	6	59	neg		
101	na	58			
102	61.25	108	neg	neg	
103	66	89	neg	neg	
104	76.5	71			
**1 heterozygous mutation in NPC1**					
105	12	287	neg		

Supplementary table S4 Primer sequences of NPC1, NPC2 (gDNA and transcript) and SMPD1 (gDNA)

**NPC1 gDNA**	
Exon 1	For AAC AGC CCG GGG AAG TAG<br>Rev TTC TGG TGC CAC ATC CTA CA
Exon 2	For TCA CTG ACT TGA CAA ATA GGC ATT<br>Rev AAG CCT TTG GAG TTA CGG TGT
Exon 3	For TTG TCA GGA GAG GAG AGA AAG C<br>Rev ACA CAC ACG CAT GCA TAC AC
Exon 4	For AAG TGC AGA ATT CAG AGA AGC A<br>Rev AGT TCA AGT CCT AGG ATA CAA GCA A
Exon 5	For TTG CAG GGT GAT AAG CCA AT<br>Rev AGA GAT GGG GCC TCA CTA CA
Exon 6	For TTT CAG TGG GCT TTT CTT TGA<br>Rev AAG AAT CAG TGG CTA ATG CAC A
Exon 7/8	For AAA TTT TCT CCT GAG ACTG GTG A<br>Rev AAC GCC ACT GAA GTC TTC TTT C
Exon 9	For CCT CAG GGC AAT GCT GAT TA<br>Rev TGT TGT TTG CTC ACC TCT GG
Exon 10	For GGC CCA TGT TGT CCT TAG AA<br>Rev TGG CTA ACC CAA TCC ACT TC
Exon 11	For AGA GCC CAG AGA TAC AGT CCA<br>Rev GCA CCT GCT CAA AGG TAA ATG
Exon 12/13	For GCT GGG GCA GGA GAA GCA C<br>Rev GGT CCT AAG ATG TCC ACA GC
Exon 13/14	For TGT TGC CCG AGC AGC CCT AG<br>Rev GGA AGC AAC ACA AAG GGA CA
Exon 15/17	For CCT GTG CTG GCT CCT TGT AT<br>Rev GTT CTG CAG CTG TGC CCA GAG
Exon 17/19	For AAT TTG GAC TCC TGG TGC TG<br>Rev TAA ACT GAG GCA CGA TGC AA
Exon 18/20	For ACC TCC TGG CAC CCT CTT AT<br>Rev GTG AAT GCC CAC GCT AAG AC
Exon 21/22	For GCC TTC TTC ATC CCT GAA AT<br>Rev AGC ACT CCT CCA GCA CTC AT
Exon 23	For CAG GGT GCC CTG GGT AAT<br>Rev CGT ACC AAC AGG TAC AGT TCC A
Exon 24	For AAA ATT AGC CAG GCA TGG TG<br>Rev CAT CAT GAA TCC CCT GGA TG
Exon 25	For GCT GAG GCA GGA AGC TTG CT<br>Rev CTG AGT TCA CAG GCG CTA CG
IVS9	For TTC CTC ATG GGG TTC TTA CG<br>Rev AAA TAC AGC CCA GTG CCA TC
**NPC2 gDNA**	
Exon 1	For GTG ACA GGT CGC CTG ACT G<br>Rev CAA AAC ACG CGT TCT AAG GA
Exon 2	For TTC CAT CCT GGG AAA CAG TC<br>Rev GGC ACA GTG AAC CCT AGC TT
Exon 3	For TGT TTG GAG TGA ATG CTT GC<br>Rev GCC TAA CAC CGC ACC TAT CT
Exon 4/5	For ACA TGC TAA GCA GCC CTC AT<br>Rev AGC ACC TCC TCT TCA ACG AA

NPC1 transcript	
Fragment I	For GTC AGC GCC TGC TCC TGC TC<br>Rev TGC CTG TCC ATT GTC CTT AT
Fragment II	For GTA CAA TGC CTG CCG GGA TG<br>Rev CAA GCG GAG GTC CAA AGG GT
Fragment III	For GCG TGT TCG TCA GGC CTG GT<br>Rev GCA CGA TCA AGA TGC CCG CG
Fragment IV	For CTC CAG AGG GCC CAG GCC TG<br>Rev ACA GGC GGA CCC GCA TGC AG
Fragment V	For CGT CCA GGC CTC AGA GAG CT<br>Rev TAT CGC GCC CAG GGA CAC AC
Fragment VI	For GTC GGA GCC ACG TAC TTC AT<br>Rev GTT CAC AGG CGC TAC GTT CA
**NPC2 transcript**	
Full length	For GAC AGG TCG CCT GAC TGG GC<br>Rev GAA CCA GCC ACC CGG AGC TC
**SMPD1 gDNA**	
Exon 1	For CAG TCA GCC GAC TAC AGA GA<br>Rev AGG TCA CAA TGG GGT GAT GG
Exon 2I*	For GCA AAG GTG TGC ACT GAG CT<br>Rev GGG TCT GCA CAG TCA GGG TC
Exon 2II*	For GCA TCC TCT TCC TCA CTG AC<br>Rev AGG GGA GCC AAA TGA AGA GC
Exon 3/4	For TGT GAG CTC CTT GCA GGT GG<br>Rev CAT GGG ACA ACA GGG ATG GT
Exon 5/6	For TCC CTG TTG TCC CAT GGA GT<br>Rev GCC CAG GGA CTG GTT CTT TC
*due to the size of exon 2, two fragments were amplified (I and II)	

References

1 Greer WL, Dobson MJ, Girouard GS, Byers DM, Riddell DC, Neumann PE. Mutations in NPC1 Highlight a Conserved NPC1-Specific Cysteine-Rich Domain. *Am J Hum Genet* 1999; **65**: 1252–60.

2 Saito Y, Suzuki K, Nanba E, Yamamoto T, Ohno K, Murayama S. Niemann–Pick type C disease: Accelerated neurofibrillary tangle formation and amyloid β deposition associated with apolipoprotein E ε4 homozygosity. *Ann Neurol* 2002; **52**: 351–5.

3 Meiner V, Shpitzen S, Mandel H, *et al.* Clinical-biochemical correlation in molecularly characterized patients with Niemann-Pick type C. *Genet Med* 2001; **3**: 343–8.

4 Millat G, Marcais C, Rafi MA, *et al.* Niemann-Pick C1 Disease: The I1061T Substitution Is a Frequent Mutant Allele in Patients of Western European Descent and Correlates with a Classic Juvenile Phenotype. *Am J Hum Genet* 1999; **65**: 1321–9.

5 Yamamoto T, Nanba E, Ninomiya H, *et al.* NPC1 gene mutations in Japanese patients with Niemann-Pick disease type C. *Hum Genet* 1999; **105**: 10–6.

6 Fancello T, Dardis A, Rosano C, *et al.* Molecular analysis of NPC1 and NPC2 gene in 34 Niemann–Pick C Italian Patients: identification and structural modeling of novel mutations. *neurogenetics* 2009; **10**: 229–39.

7 Watari H, Blanchette-Mackie EJ, Dwyer NK, *et al.* Niemann-Pick C1 protein: Obligatory roles for N-terminal domains and lysosomal targeting in cholesterol mobilization. *Proc Natl Acad Sci U S A* 1999; **96**: 805–10.

8 Carstea ED, Morris JA, Coleman KG, *et al.* Niemann-Pick C1 Disease Gene: Homology to Mediators of Cholesterol Homeostasis. *Science* 1997; **277**: 228–31.

9 Ribeiro I, Marcão A, Amaral O, Miranda MS, Vanier MT, Millat G. Niemann-Pick type C disease: NPC1 mutations associated with severe and mild cellular cholesterol trafficking alterations. *Hum Genet* 2001; **109**: 24–32.

10 Sun X, Marks DL, Park WD, *et al.* Niemann-Pick C Variant Detection by Altered Sphingolipid Trafficking and Correlation with Mutations within a Specific Domain of NPC1. *Am J Hum Genet* 2001; **68**: 1361–72.

11 Garver WS, Jelinek D, Meaney FJ, *et al.* The National Niemann-Pick Type C1 Disease Database: correlation of lipid profiles, mutations, and biochemical phenotypes. *J Lipid Res* 2010; **51**: 406–15.

12 Park WD, O'Brien JF, Lundquist PA, *et al.* Identification of 58 novel mutations in Niemann-Pick disease type C: Correlation with biochemical phenotype and importance of PTC1-like domains in NPC1. *Hum Mutat* 2003; **22**: 313–25.

13 Rodríguez-Pascau L, Coll MJ, Vilageliu L, Grinberg D. Antisense oligonucleotide treatment for a pseudoexon-generating mutation in the NPC1 gene causing Niemann-Pick type C diseaseb. *Hum Mutat* 2009; **30**: E993–1001.

14 Greer WL, Riddell DC, Gillan TL, *et al.* The Nova Scotia (Type D) Form of Niemann-Pick Disease Is Caused by a G3097→T Transversion in NPC1. *Am J Hum Genet* 1998; **63**: 52–4.

15 Millat G, Marcais C, Tomasetto C, *et al.* Niemann-Pick C1 Disease: Correlations between NPC1 Mutations, Levels of NPC1 Protein, and Phenotypes Emphasize the Functional Significance of the

Putative Sterol-Sensing Domain and of the Cysteine-Rich Luminal Loop. *Am J Hum Genet* 2001; **68**: 1373–85.

16 Stampfer M, Theiss S, Amraoui Y, *et al.* Niemann-Pick disease type C clinical database: cognitive and coordination deficits are early disease indicators. *Orphanet J Rare Dis* 2013; **8**: 35.

17 Fernandez-Valero E, Ballart A, Iturriaga C, *et al.* Identification of 25 new mutations in 40 unrelated Spanish Niemann-Pick type C patients: genotype-phenotype correlations. *Clin Genet* 2005; **68**: 245–54.

18 Yamamoto T, Ninomiya H, Matsumoto M, *et al.* Genotype-phenotype relationship of Niemann-Pick disease type C: a possible correlation between clinical onset and levels of NPC1 protein in isolated skin fibroblasts. *J Med Genet* 2000; **37**: 707–12.

19 Yang C-C, Su Y-N, Chiou P-C, *et al.* Six novel NPC1 mutations in Chinese patients with Niemann–Pick disease type C. *J Neurol Neurosurg Psychiatry* 2005; **76**: 592–5.

20 Kaminski WE, Klünemann HH, Ibach B, Aslanidis C, Klein HE, Schmitz G. Identification of novel mutations in the NPC1 gene in German patients with Niemann-Pick C disease. *J Inherit Metab Dis* 2002; **25**: 385–9.

21 Tarugi P, Ballarini G, Bembi B, *et al.* Niemann-Pick type C disease mutations of NPC1 gene and evidence of abnormal expression of some mutant alleles in fibroblasts. *J Lipid Res* 2002; **43**: 1908–19.

22 Millat G, Chikh K, Naureckiene S, *et al.* Niemann-Pick Disease Type C: Spectrum of HE1 Mutations and Genotype/Phenotype Correlations in the NPC2 Group. *Am J Hum Genet* 2001; **69**: 1013–21.

23 Griese M, Brasch F, Aldana V, *et al.* Respiratory disease in Niemann-Pick type C2 is caused by pulmonary alveolar proteinosis. *Clin Genet* 2010; **77**: 119–30.

24 Zhang H, Wang Y, Lin N, *et al.* Diagnosis of Niemann-Pick disease type C with 7-ketocholesterol screening followed by NPC1/NPC2 gene mutation confirmation in Chinese patients. *Orphanet J Rare Dis* 2014; **9**: 82.

25 Zech M, Nübling G, Castrop F, *et al.* Niemann-Pick C Disease Gene Mutations and Age-Related Neurodegenerative Disorders. *PLoS ONE* 2013; **8**. DOI:10.1371/journal.pone.0082879.

26 Bauer P, Balding DJ, Klünemann HH, *et al.* Genetic screening for Niemann-Pick disease type C in adults with neurological and psychiatric symptoms: findings from the ZOOM study. *Hum Mol Genet* 2013; **22**: 4349–56.

27 Takahashi T, Suchi M, Desnick RJ, Takada G, Schuchman EH. Identification and expression of five mutations in the human acid sphingomyelinase gene causing types A and B Niemann-Pick disease. Molecular evidence for genetic heterogeneity in the neuronopathic and non-neuronopathic forms. *J Biol Chem* 1992; **267**: 12552–8.

28 Rodríguez-Pascau L, Gort L, Schuchman EH, Vilageliu L, Grinberg D, Chabás A. Identification and characterization of SMPD1 mutations causing Niemann-Pick types A and B in Spanish patients. *Hum Mutat* 2009; **30**: 1117–22.

29 Simonaro CM, Desnick RJ, McGovern MM, Wasserstein MP, Schuchman EH. The Demographics and Distribution of Type B Niemann-Pick Disease: Novel Mutations Lead to New Genotype/Phenotype Correlations. *Am J Hum Genet* 2002; **71**: 1413–9.

30 Takahashi T, Suchi M, Sato W, *et al.* Identification and expression of a missense mutation (Y446C) in the acid sphingomyelinase gene from a Japanese patient with type A Niemann-Pick disease. *Tohoku J Exp Med* 1995; **177**: 117–23.

31 Pavlů H, Elleder M. Two novel mutations in patients with atypical phenotypes of acid sphingomyelinase deficiency. *J Inherit Metab Dis* 1997; **20**: 615–6.
